# Modulation of sunflower growth via regulation of antioxidants, oil content and gas exchange by arbuscular mycorrhizal fungi and quantum dot biochar under chromium stress

**DOI:** 10.1186/s12870-023-04637-6

**Published:** 2023-12-08

**Authors:** Musarrat Ramzan, Talha Jamshaid, Liaqat Ali, Khadim Dawar, Rabia Saba, Usama Jamshaid, Shah Fahad, Saleh H. Salmen, Mohammad Javed Ansari, Subhan Danish, Misbah Hareem, Hina Saif, Khurrum Shahzad

**Affiliations:** 1 Department of Botany, Faculty of Chemical and Biological Sciences, The Islamia University of Bahawalpur, Bahawalpur, Pakistan; 2 Department of Pharmaceutics, Faculty of Pharmacy, The Islamia University of Bahawalpur, Bahawalpur, Punjab Pakistan; 3 Cholistan institute of Desert Studies, The Islamia University of Bahawalpur, Bahawalpur, Punjab Pakistan; 4 Department of Soil and Environmental Science, the University of Agriculture Peshawar, Peshawar, Pakistan; 5 Department of Biological Science, University of Thal Bhakkar, Bhakkar, Pakistan; 6 Faculty of Pharmacy, University Des Strasbourg, Strasbourg, France; 7 Department of Agronomy, Abdul Wali Khan University Mardan, Mardan, 23200 Khyber Pakhtunkhwa Pakistan; 8 Department of Botany and Microbiology, College of Science, King Saud University, PO Box -2455, Riyadh, 11451 Saudi Arabia; 9 Department of Botany, Hindu College Moradabad (MJP Rohilkhand University Bareilly), Moradabad, 244001 India; 10 Department of Soil Science, Faculty of Agricultural Sciences and Technology, Bahauddin Zakariya University, Multan, Punjab Pakistan; 11 Department of Environmental Sciences, The Woman University Multan, Multan, Punjab Pakistan; 12 Department of Soil Science, Water and Marine Sciences, Lasbela university of Agriculture, Uthal, Balochistan, Pakistan

**Keywords:** Antioxidants, Chromium, Chlorophyll content, Gas exchange, Oil content, Sunflower

## Abstract

Chromium (Cr) toxicity significantly threatens sunflower growth and productivity by interfering with enzymatic activity and generating reactive oxygen species (ROS). Zinc quantum dot biochar (ZQDB) and arbuscular mycorrhizal fungi (AMF) have become popular to resolve this issue. AMF can facilitate root growth, while biochar tends to minimize Cr mobility in soil. The current study aimed to explore AMF and ZQDB combined effects on sunflower plants in response to Cr toxicity. Four treatments were applied, i.e. NoAMF + NoZQDB, AMF + 0.40%ZQDB, AMF + 0.80%ZQDB, and AMF + 1.20%ZQDB, under different stress levels of Cr, i.e. no Cr (control), 150 and 200 mg Cr/kg soil. Results showed that AMF + 1.20%ZQDB was the treatment that caused the greatest improvement in plant height, stem diameter, head diameter, number of leaves per plant, achenes per head, 1000 achenes weight, achene yield, biological yield, transpiration rate, stomatal conductance, chlorophyll content and oleic acid, relative to the condition NoAMF + No ZQDB at 200 mg Cr/kg soil. A significant decline in peroxidase (POD), superoxide dismutase (SOD), and catalase (CAT) while improvement in ascorbate peroxidase (APx), oil content, and protein content further supported the effectiveness of AMF + 1.20%ZQDB against Cr toxicity. Our results suggest that the treatment AMF + 1.20%ZQDB can efficiently alleviate Cr stress in sunflowers.

## Introduction

Chromium (Cr) toxicity in plants refers to the harmful effects of excessive amounts of chromium on plant growth and development [1–5]. Cr is a naturally occurring heavy metal found in soil, water, and air [6]. While it is an essential nutrient for plants in trace amounts, high chromium concentrations can be toxic. There are two main forms of chromium: trivalent chromium (Cr(III)) and hexavalent chromium (Cr(VI)) [1]. Trivalent chromium is the less toxic form and is even considered an essential nutrient for plants, playing a role in various physiological processes [1]. On the other hand, hexavalent chromium is highly toxic to plants and poses a significant threat to their health [7]. When plants are exposed to high levels of hexavalent chromium, it can disrupt their cellular processes and lead to oxidative stress [7–9]. Hexavalent chromium can enter plant cells through the root system and accumulate in various plant tissues [1]. It can interfere with enzymatic activity, disrupt cellular membranes, and generate ROS that can damage proteins, lipids, and DNA [10].

Biofertilizers [11–13], such as zinc quantum dot biochar (ZQDB) and arbuscular mycorrhizal fungi (AMF), are two treatments that can potentially be used to mitigate chromium toxicity in plants [14–16]. AMF, beneficial soil fungi, can enhance nutrient uptake, decrease chromium uptake, and provide antioxidant activity, all of which might alleviate chromium toxicity [14]. Inoculating plants with AMF spores or using AMF-rich soil as an amendment can be effective strategies [17]. On the other hand, ZQDB, a combination of biochar [13, 18] and zinc nanoparticles [19], can facilitate processes such as heavy metal adsorption, immobilization and ion exchange in soil. Such processes facilitate in increasing plant tolerance towards heavy metal toxicity [16]. The biochar matrix stabilizes the quantum dots and facilitates controlled release in the soil [20]. However, further research and field trials are needed to optimize the application methods and assess the efficacy of these treatments in different soil and plant conditions.

Sunflower (*Helianthus annuus*) belongs to the family Asteraceae and are commercially cultivated for their valuable seeds, a rich source of edible oil. Sunflower oil is widely consumed worldwide and is a popular choice for cooking oil due to its favourable fatty acid profile and nutritional benefits [21]. The oil extracted from sunflower seeds is used in culinary applications, food processing, and the production of margarine and mayonnaise. The sunflower industry contributes significantly to the global edible oil market, and it plays a crucial role in the agricultural economy of many countries [22–25].

While AMF and biochar have been individually studied for their effects on heavy metal tolerance and remediation, their combined application, specifically targeting chromium toxicity in sunflowers, is relatively unexplored. Using ZQDB with AMF to alleviate chromium toxicity is a novel approach. Our study aims to explore and test the combined effect of AMF and ZQDB on Cr toxicity mitigation in crops, and specifically in sunflowers.

## Materials and methods

### Experimental site and climatic condition

**Fig. 1:**
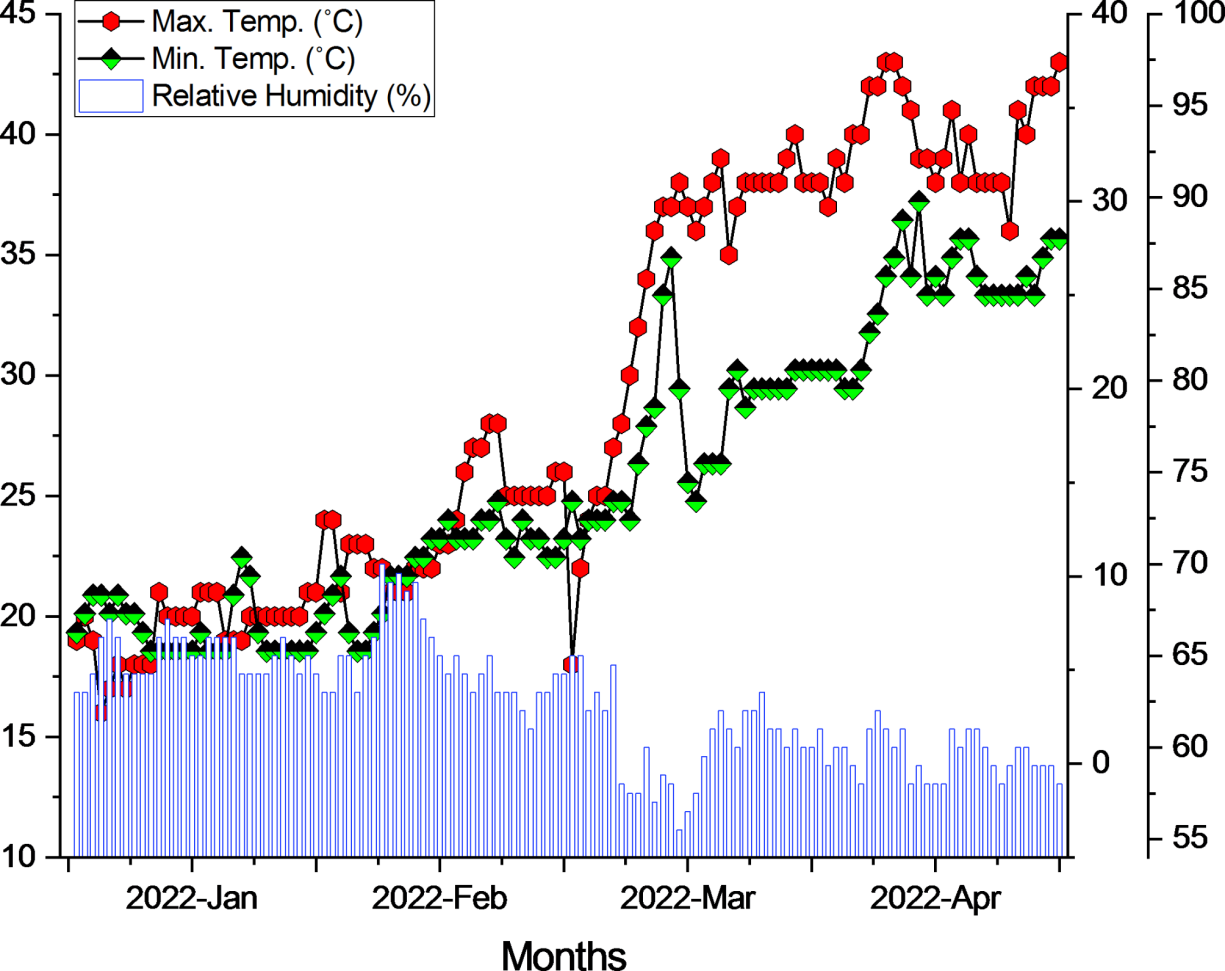
Experimental site climatic data

In 2022, a pot experiment was conducted at the research area of the Faculty of Agricultural Sciences and Technology, Bahauddin Zakariya University in Multan, Punjab, Pakistan (30°15′49″N 71°30′35″E). The climate in the research area was arid. The climatic data collected during the experiment is presented in Fig. 1. The soil used for the experiment was collected from the Agricultural Research Farm of Bahauddin Zakariya University, Multan. The physicochemical properties of soil are provided in Table 1.

### Arbuscular mycorrhizal fungi (AMF)

AMF into the soil, we acquired a commercial inoculum known as Clonex® Root Maximizer. This inoculum primarily contained Glomus species and had an estimated propagule count of 158 per gram. To facilitate effective colonization, we mixed 2.5 g of the inoculum with every 5 kg of soil. Subsequently, this soil-inoculum mixture was applied for the purpose of inoculating the research soil as per the treatment plan [24].

### Zinc quantum dot biochar

To synthesize zinc (Zn) quantum dots, we followed a modified procedure based on the acid-base reaction method originally developed by Song et al. [23]. The synthesis process commenced by combining zinc acetate dihydrate, oleic acid, and 1-octadecene in a three-neck flask, stirring the mixture at room temperature for 10 min, followed by heating at 120 °C under a nitrogen atmosphere for 30 min to achieve a clear solution. Subsequently, a sodium hydroxide solution, prepared by dissolving sodium hydroxide in methanol, was added to the flask containing the zinc-oleic acid mixture, further heated for 2 h at 120 °C. The addition of hexane precipitated the formation of zinc quantum dots (QDs), which were isolated through centrifugation and purified using ethanol, ultimately suspended in toluene. These QDs were then combined with biochar at a ratio of 1:100 in a separate container and stirred for 24 h, facilitating the binding of QDs with the biochar substrate. Post-binding, the Zn-quantum dot biochar (ZQDB) mixture underwent multiple ethanol washes to eliminate unbound QDs. Finally, the ZQDB mixture was dried in a vacuum oven at 60 °C for 24 h to remove residual moisture, ensuring the stability and purity of the resulting composite material. The characteristics of ZQDB are provided in Table 1.

**Table 1:** Pre-experimental biochar and irrigation characteristics

Soil	Values	Biochar	Values	Irrigation	Values
pH	8.63	pH	8.45	pH	6.75
ECe (dS/m)	3.69	ECe (dS/m)	4.35	EC (µS/cm)	205
SOC (%)	0.55	Volatile Matter (%)	18.00	Carbonates (meq./L)	0.00
TN (%)	0.032	Ash Content (%)	33.00	Bicarbonates (meq./L)	6.12
AK (mg/kg)	121	Fixed carbon (%)	49.00	Chloride (meq./L)	0.15
EP (mg/kg)	4.23	TN (%)	0.15	Ca + Mg (meq./L)	4.00
Sand (%)	25.00	TK (%)	1.41	Sodium (mg/L)	199.00
Silt (%)	40.00	TP (%)	0.65	TN = Total Nitrogen EP = Extractable Phosphorus AK = Available Potassium CEC = Cation Exchange Capacity EC = Electrical Conductivity	
Clay (%)	35.00	Surface area (m²/g)	350		
Texture	Clay Loam	CEC (meq./100 g)	411		

### Seeds collection and sterilization

The sunflower seeds utilized in this research were procured from a certified seed dealer officially recognized by the Government of Punjab, Pakistan. Prior to sowing, the seeds underwent a surface sterilization process involving treatment with a 5% sodium hypochlorite solution, followed by three consecutive rinses using 95% ethanol. To ensure the elimination of residual sterilizing agents, the seeds were washed three times with sterilized deionized water [25]. The sowing was done on 15th Jan, 2022.

### Treatment plan

The control group served as the baseline, with no additional treatments applied. In the control group, neither AMF nor zinc quantum dots (ZQDB) were added (NoAMF + NoZQDB). Treatments involved the inoculation of AMF in combination with different concentrations of ZQDB: 0.40%, 0.80%, and 1.20%. Additionally, three distinct Cr stress levels were applied, labeled as control (0Cr), 150Cr (150 mg Cr/kg soil), and 200Cr (200 mg Cr/kg soil) [24]. All the treatments were applied in a completely randomized design (CRD) in 4 replicates.

### Fertilizer

To meet the nutritional requirements of the plants, a recommended dose of nitrogen (N), phosphorus (P), and potassium (K) at a ratio of 60:40:25 kg/acre (N = 1.110 g, P = 0.7425 g and K = 0.1875 g per pot) was applied. Various sources of artificial fertilizers were used to supply these essential nutrients. The application of fertilizers was done at different stages of the plant’s growth, including sowing, the first and second irrigation, and the flowering stage, with varying proportions. 

### Irrigation

Each pot’s irrigation was maintained using a moisture meter (ADVANCED™; 4 in 1 Soil Meter; China). The irrigation was provided by monitoring daily to maintain the scale of the instrument Wet = 70% field capacity.

### Harvesting and data collection

After 115 days of sowing, plants were harvested. Ten plants were randomly chosen from each plot for data collection to evaluate the impact of different treatments on plant growth and development. Several parameters, namely plant height, number of leaves per plant, stem diameter, and head diameter, were measured for each selected plant. Three samples, each containing 1000 achenes, were collected from every plot to determine the achene count. Additionally, the weight of 1000 achenes was measured using an electric balance. The entire plot was harvested, and the plants were dried naturally using sunlight. The total biomass comprising the stems and achenes was determined by weighing with a spring balance and then converted to kilograms per hectare (kg/ha). The heads were separated from the plants, and the grains were manually threshed. The weight of all the grains obtained from each plot was measured using an electric balance and expressed in kilograms per hectare (kg/ha).

### Chlorophyll contents

A non-destructive method involving a SPAD meter (SPAD-502 Chlorophyll Index, SCI) was employed to assess the chlorophyll content of the plants.

### Gas exchange attributes

To assess gas exchange parameters, we employed an Infra-Red Gas Analyzer (CI-340 Photosynthesis system, CID, Inc. USA) for the determination of transpiration rate and stomatal conductance (gs).

### Antioxidants

To obtain antioxidant enzymes from fresh plant tissue, we utilized a chilled 100 mM phosphate buffer (pH 7.8) containing polyvinyl pyrrolidine (PVP, 0.1%) and ethylenediaminetetraacetic acid (EDTA, 0.5 mM). The method of [26] was used to measure the activity of ascorbate peroxidase (APx). For the extraction of APX, the extraction buffer was supplemented with 2 mM ascorbate. The assessment of superoxide dismutase (SOD) activity involved measuring the photochemical reduction of nitroblue tetrazolium (NBT) at 560 nm [27]. We assessed catalase (CAT) enzyme activity by monitoring absorbance at 240 nm for a duration of 2 min, with H_2_O_2_ serving as the substrate [28]. Additionally, peroxidase (POD) activity was determined following a specific assay method [29]. The reduced glutathione (GSH) content was estimated using the method described by [30]. Hydrogen peroxide (H_2_O_2_) concentration was determined using a spectrophotometer at 390 nm [31].

### Oil contents

The oil content of intact seeds was determined using nuclear magnetic resonance (NMR) with the MQC23 instrument manufactured by Oxford Instruments, UK. A standard sample containing 5 g of seeds from various sunflower hybrids with known oil content was used to calibrate the instrument. The analysis of fatty acid profiles, including stearic acid, palmitic acid, linolenic acid, and oleic acid, was conducted using near-infrared spectroscopy (NIRS) [32].

### Statistical analysis

A standard statistical procedure was followed for the statistical analysis. The means were compared using Fisher’s LSD test, with a significance level of *p* ≤ 0.05. To aid visualization, cluster plot convex hull and hierarchical cluster plot techniques were employed using OriginPro software [33].

## Results

### Plant height, stem diameter, head diameter, and number of leaves per plant

**Fig. 2:**
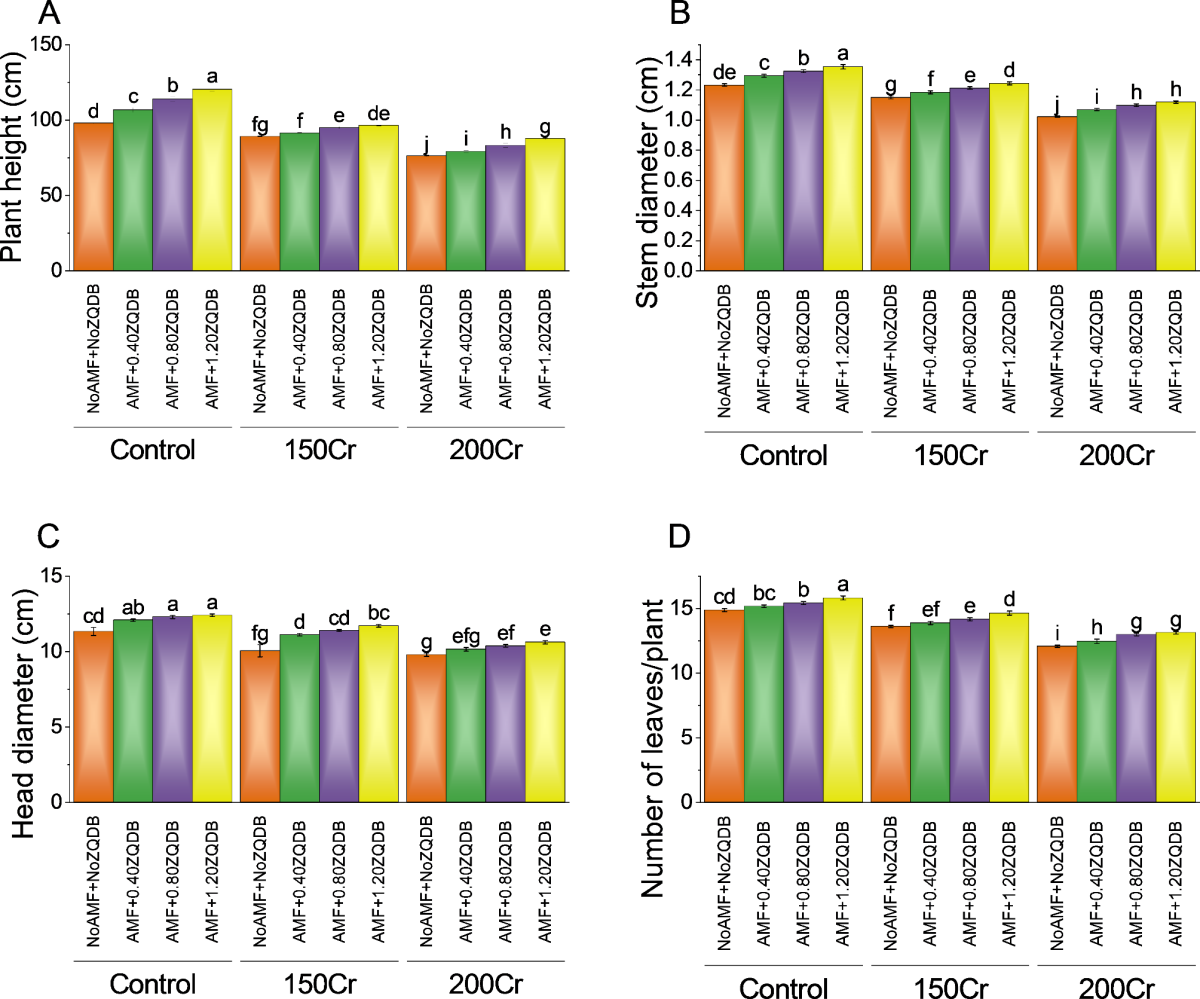
Effect of treatments on plant height (**A**), stem diameter (**B**), head diameter (**C**), and number of leaves/plant (**D**) of sunflower cultivated under normal conditions (control) and chromium (Cr) toxicity, i.e., 150 mg Cr/ kg soil (150Cr) and 200 mg Cr/ kg soil (200Cr). Bars are means of 4 replicates ± SE. Treatments were compared using Fisher’s LSD; p ≤ 0.05. Different letters on the bars showed significant changes. AMF = Arbuscular mycorrhizae fungi; ZQDB = zinc quantum dot biochar

The control group without any stress treatment had a plant height of 98 cm. The application of AMF + 0.40ZQDB increased the plant height to 106.75 cm, representing a percentage change of ~ 9%. Further increase in the stress treatment to AMF + 0.80ZQDB led to a plant height of 114 cm, resulting in a percentage change of ~ 16%. The highest stress treatment of AMF + 1.20ZQDB resulted in a plant height of 120.5 cm, indicating a percentage change of ~ 23%. Additionally, the study investigated the effect of amendments application on two other stress concentrations, 150Cr and 200Cr. For the 150Cr concentration, the control group (NoAMF + NoZQDB) had a plant height of 89.25 cm. When treated with AMF + 0.40ZQDB, the plant height slightly increased to 91.5 cm, representing a percentage change of ~ 3%. A further increase in stress treatment to AMF + 0.80ZQDB resulted in a plant height of 95 cm, indicating a percentage change of ~ 6%. The highest stress treatment of AMF + 1.20ZQDB for the 150Cr concentration led to a plant height of 96.5 cm, representing a percentage change of ~ 8%. The highest stress treatment of AMF + 1.20ZQDB for the 200Cr concentration resulted in a plant height of 87.75 cm, indicating a percentage change of ~ 15% (Fig. 2A).

Treatment with AMF + 0.40ZQDB led to a slightly increased stem diameter of 1.29 cm, representing a percentage change of ~ 5%. Further intensifying the stress treatment to AMF + 0.80ZQDB resulted in a stem diameter of 1.32 cm, indicating a percentage change of ~ 8%. The highest stress treatment of AMF + 1.20ZQDB showed the maximum effect on stem diameter, with a value of 1.35 cm and a percentage change of ~ 10%. Moreover, the study examined the impact of amendments on two additional stress concentrations: 150Cr and 200Cr. The control group for the 150Cr concentration (NoAMF + NoZQDB) displayed a stem diameter of 1.15 cm. When treated with AMF + 0.40ZQDB, the stem diameter increased slightly to 1.18 cm, representing a percentage change of ~ 3%. Further stress treatment with AMF + 0.80ZQDB resulted in a stem diameter of 1.21 cm, indicating a percentage change of ~ 5%. The highest stress treatment of AMF + 1.20ZQDB for the 150Cr concentration led to a stem diameter of 1.24 cm, representing a percentage change of ~ 8%. For the 200Cr concentration, the control group exhibited a stem diameter of 1.02 cm. Treatment with AMF + 0.40ZQDB resulted in a slightly increased stem diameter of 1.06 cm, indicating a percentage change of ~ 5%. Further stress treatment to AMF + 0.80ZQDB led to a stem diameter of 1.09 cm, representing a percentage change of ~ 7%. The highest stress treatment of AMF + 1.20ZQDB for the 200Cr concentration resulted in a stem diameter of 1.11 cm, indicating a percentage change of ~ 9% (Fig. 2B).

The control exhibited a head diameter of 11.34 cm. Treatment with AMF + 0.40ZQDB resulted in an increased head diameter of 12.09 cm, representing a percentage change of ~ 7%. Further intensifying the stress treatment to AMF + 0.80ZQDB led to a head diameter of 12.29 cm, indicating a percentage change of ~ 9%. The highest stress treatment of AMF + 1.20ZQDB showed the maximum effect on head diameter, with a value of 12.41 cm and a percentage change of ~ 10%. Additionally, the study investigated the impact of amendments application on two additional stress concentrations: 150Cr and 200Cr. The control group for the 150Cr concentration (NoAMF + NoZQDB) displayed a head diameter of 10.06 cm. When treated with AMF + 0.40ZQDB, the head diameter increased to 11.12 cm, representing a percentage change of ~ 11%. Further stress treatment with AMF + 0.80ZQDB resulted in a head diameter of 11.40 cm, indicating a percentage change of ~ 13. The highest stress treatment of AMF + 1.20ZQDB for the 150Cr concentration led to a head diameter of 11.71 cm, representing a percentage change of ~ 17. For the 200Cr concentration, the control group exhibited a head diameter of 9.78 cm. Treatment with AMF + 0.40ZQDB resulted in a head diameter of 10.15 cm, indicating a percentage change of ~ 4% (Fig. 2C).

In the control treatment, the number of leaves per plant was 14.88. Treatment with AMF + 0.40ZQDB led to a slightly increased of 15.17 leaves per plant, resulting in a percentage change of ~ 2%. Moreover, the study examined the effect of amendments application on two additional stress concentrations: 150Cr and 200Cr. The control group for the 150Cr concentration (NoAMF + NoZQDB) displayed a number of leaves per plant of 13.61. When treated with AMF + 0.40ZQDB, the number of leaves per plant increased slightly to 13.88, resulting in a percentage change of ~ 2%. Further stress treatment with AMF + 0.80ZQDB resulted in a of 14.17 leaves per plant, indicating a percentage change of ~ 4. The highest stress treatment of AMF + 1.20ZQDB for the 150Cr concentration led to a of 14.65 leaves per plant, representing a percentage change of ~ 8. For the 200Cr concentration, the control group exhibited a of 12.07 leaves per plant. Treatment with AMF + 0.40ZQDB resulted in a of 12.46 leaves per plant, indicating a percentage change of ~ 3. Further stress treatment to AMF + 0.80ZQDB led to a of 12.99 leaves per plant, representing a percentage change of ~ 8. The highest stress treatment of AMF + 1.20ZQDB for the 200Cr concentration resulted in a of 13.13 leaves per plant, indicating a percentage change of ~ 9 (Fig. 2D).

### Achenes per head, 1000 achenes weight, achene yield, and biological yield

**Fig. 3:**
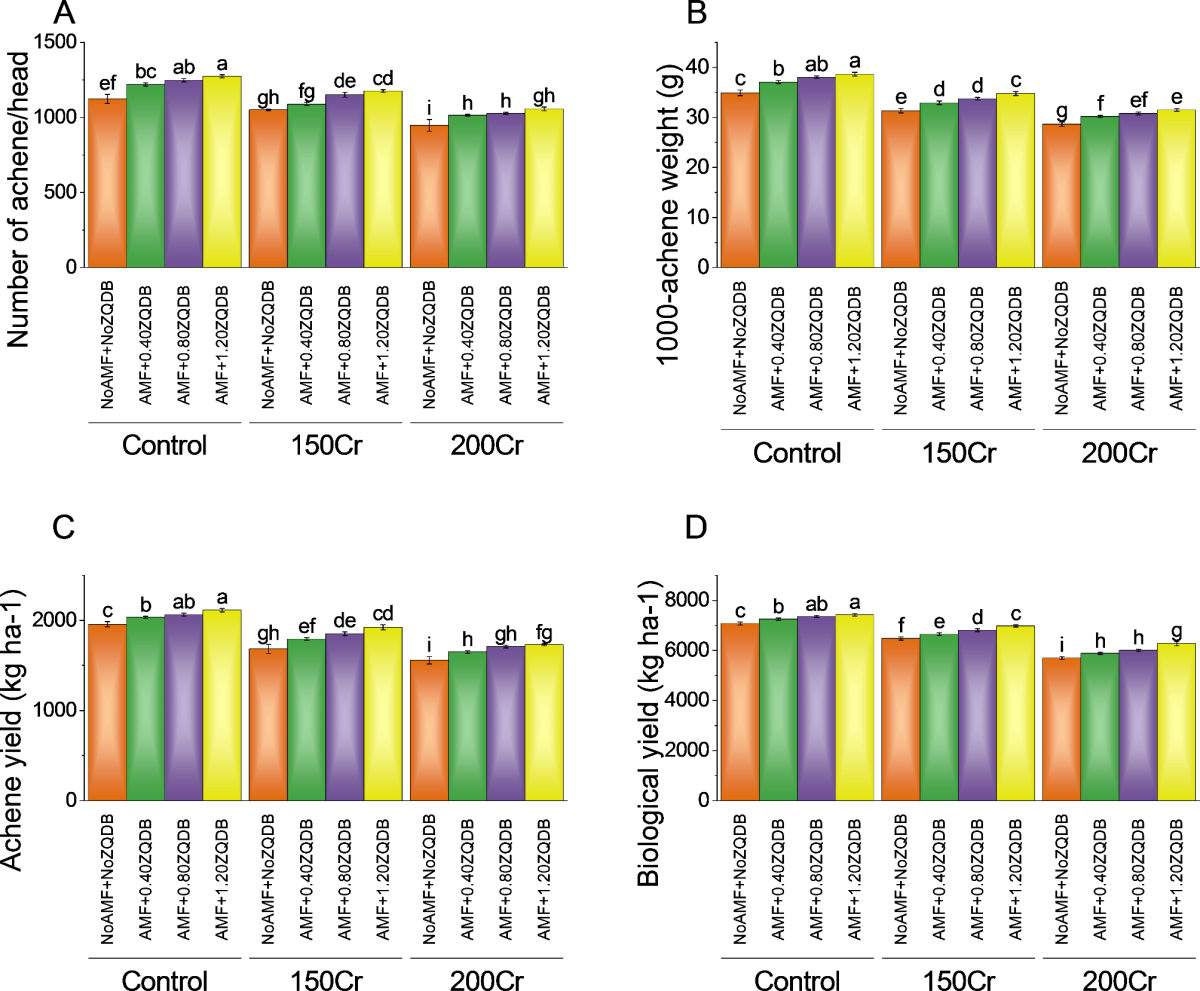
Effect of treatments on the number of achene/head (**A**), 1000-achene weight (**B**), achene yield (**C**), and biological yield (**D**) of sunflower cultivated under normal conditions and Cr toxicity, i.e., 150 mg Cr/ kg soil (150Cr) and 200 mg Cr/ kg soil (200Cr). Bars are means of 4 replicates ± SE. Treatments were compared using Fisher’s LSD; p ≤ 0.05. Different letters on the bars showed significant changes. AMF = Arbuscular mycorrhizae fungi; ZQDB = zinc quantum dot biochar

Treatment with AMF + 0.40ZQDB resulted in a higher of 1219.68 achenes per head, representing a percentage change of ~ 9%. Intensifying the stress treatment to AMF + 0.80ZQDB led to a of 1247.74 achenes per head, indicating a percentage change of ~ 11. The highest stress treatment, AMF + 1.20ZQDB, exhibited the most substantial impact on the number of achenes per head, with a of 1273.82 achenes, corresponding to a percentage change of ~ 14. Furthermore, the study examined the effect of amendments application on two additional stress concentrations: 150Cr and 200Cr. The control group for the 150Cr concentration (NoAMF + NoZQDB) displayed a number of achenes per head of 1050.54. When treated with AMF + 0.40ZQDB, the increased to 1088.61 achenes per head, resulting in a percentage change of ~ 4%. Further stress treatment with AMF + 0.80ZQDB resulted in a of 1151.24 achenes per head, indicating a percentage change of ~ 10%. The highest stress treatment of AMF + 1.20ZQDB for the 150Cr concentration led to a of 1176.33 achenes per head, representing a percentage change of ~ 12%. For the 200Cr concentration, the control group exhibited a of 947.21 achenes per head. Further stress treatment with AMF + 0.80ZQDB led to a of 1027.64 achenes per head, representing a percentage change of ~ 9%. The highest stress treatment of AMF + 1.20ZQDB for the 200Cr concentration resulted in a of 1056.78 achenes per head, indicating a percentage change of ~ 12% (Fig. 3A).

In the control group, which did not undergo any stress treatment, the weight of 1000 achenes was 34.90 g. Treatment with AMF + 0.40ZQDB resulted in a slightly increased weight of 37.07 g, corresponding to a percentage change of ~ 6%. Intensifying the stress treatment to AMF + 0.80ZQDB led to a weight of 38.01 g, indicating a percentage change of ~ 9%. The highest stress treatment, AMF + 1.20ZQDB, showed the greatest impact on the weight of 1000 achenes, with a of 38.62 g, representing a percentage change of ~ 11%. Additionally, the study investigated the impact of amendments application on two additional stress concentrations: 150Cr and 200Cr. The control group for the 150Cr concentration (NoAMF + NoZQDB) exhibited a weight of 31.32 g for 1000 achenes. When treated with AMF + 0.40ZQDB, the weight increased to 32.91 g, resulting in a percentage change of ~ 5%. Further stress treatment with AMF + 0.80ZQDB resulted in a weight of 33.73 g, indicating a percentage change of ~ 8%. The highest stress treatment of AMF + 1.20ZQDB for the 150Cr concentration led to a weight of 34.77 g, representing a percentage change of 11.00%. For the 200Cr concentration, the control group displayed a weight of 28.64 g for 1000 achenes. The highest stress treatment of AMF + 1.20ZQDB for the 200Cr concentration resulted in a weight of 31.49 g, indicating a percentage change of ~ 10% (Fig. 3B).

Treatment with AMF + 0.40ZQDB resulted in a slight increase in yield to 2034.29 kg/ha, corresponding to a percentage change of ~ 4%. Intensifying the stress treatment to AMF + 0.80ZQDB further enhanced the yield to 2064.77 kg/ha, reflecting a percentage change of ~ 6%. Notably, the highest stress treatment, AMF + 1.20ZQDB, exhibited the greatest impact on achene yield, with a of 2114.46 kg/ha, representing a percentage change of ~ 8%. Moreover, the investigation extended to two additional stress concentrations, namely 150Cr and 200Cr. The control group for the 150Cr concentration (NoAMF + NoZQDB) demonstrated a achene yield of 1682.34 kg/ha. Treatment with AMF + 0.40ZQDB resulted in an increased yield of 1793.06 kg/ha, reflecting a percentage change of ~ 7%. Further stress treatment with AMF + 0.80ZQDB led to a yield of 1850.75 kg/ha, indicating a percentage change of ~ 10%. The highest stress treatment, AMF + 1.20ZQDB, for the 150Cr concentration yielded a of 1922.57 kg/ha, exhibiting a percentage change of ~ 14%. For the 200Cr concentration, the control group exhibited a achene yield of 1558.35 kg/ha. Treatment with AMF + 0.40ZQDB resulted in a yield of 1648.81 kg/ha, signifying a percentage change of ~ 6%. Further stress treatment with AMF + 0.80ZQDB led to a yield of 1709.30 kg/ha, indicating a percentage change of ~ 10%. The highest stress treatment, AMF + 1.20ZQDB, for the 200Cr concentration yielded a of 1729.90 kg/ha, representing a percentage change of ~ 11% (Fig. 3C).

The control group, which received no stress treatment (NoAMF + NoZQDB), exhibited a biological yield of 7076.73 kg/ha. Treatment with AMF + 0.40ZQDB led to a slight increase in the yield to 7246.34 kg/ha, representing a percentage change of ~ 2%. Further intensifying the stress treatment to AMF + 0.80ZQDB resulted in a yield of 7358.49 kg/ha, indicating a percentage change of ~ 4%. The highest stress treatment, AMF + 1.20ZQDB, showed the most significant impact on biological yield, yielding a of 7426.50 kg/ha, corresponding to a percentage change of ~ 5%. Moreover, the study investigated the impact of amendments application on two additional stress concentrations: 150Cr and 200Cr. The control group for the 150Cr concentration (NoAMF + NoZQDB) displayed a biological yield of 6476.63 kg/ha. Treatment with AMF + 0.40ZQDB resulted in a yield of 6647.62 kg/ha, reflecting a percentage change of ~ 3%. Further stress treatment with AMF + 0.80ZQDB led to a yield of 6810.94 kg/ha, signifying a percentage change of ~ 5%. Further stress treatment with AMF + 0.80ZQDB led to a yield of 5999.88 kg/ha, signifying a percentage change of ~ 5%. The highest stress treatment, AMF + 1.20ZQDB, for the 200Cr concentration yielded a of 6268.22 kg/ha, representing a percentage change of ~ 10% (Fig. 3D).

### Gas exchange attributes, chlorophyll contents, and electrolyte leakage

**Fig. 4:**
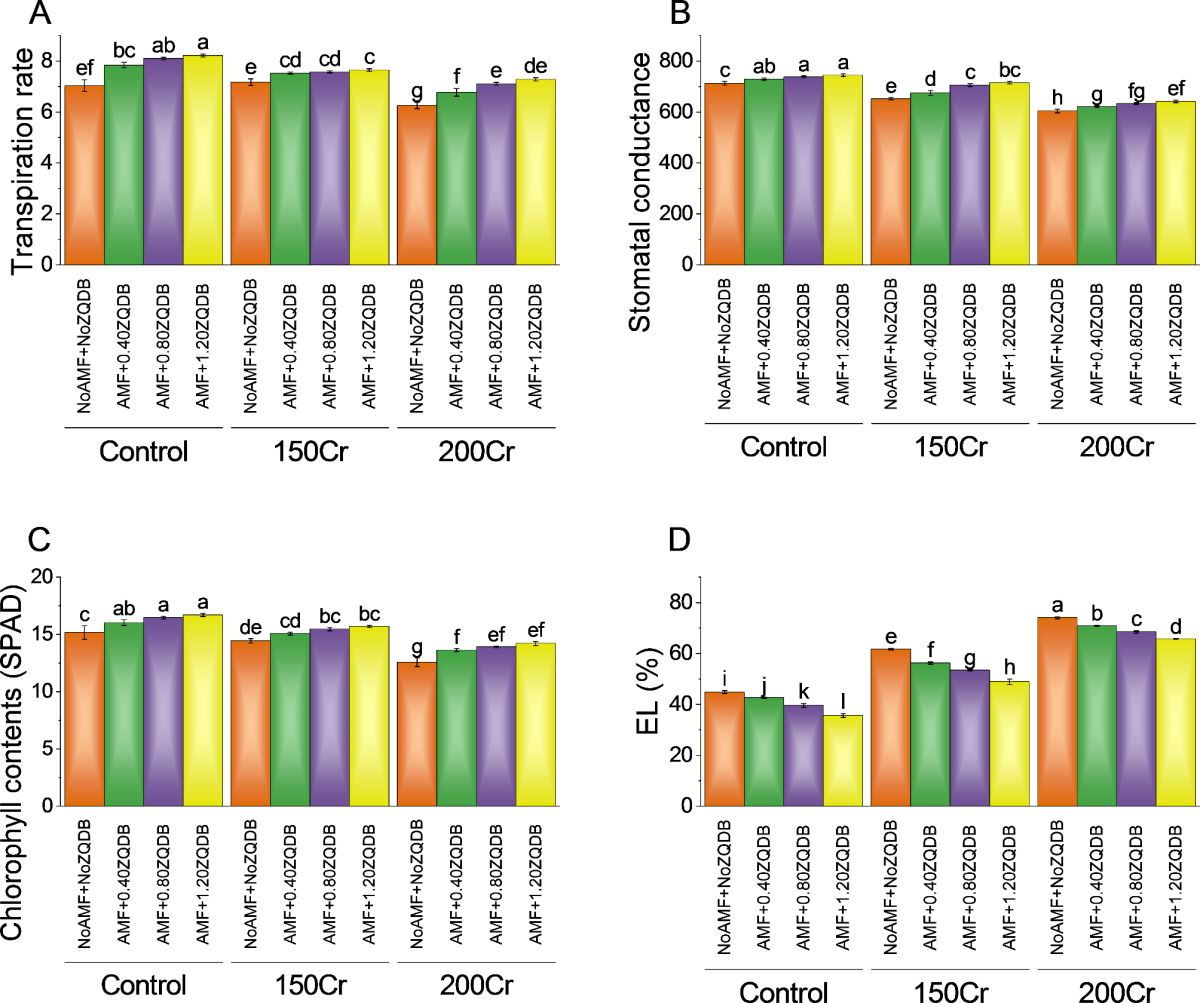
Effect of treatments on number of transpiration rate (**A**), stomatal conductance (**B**), chlorophyll contents (**C**), and electrolyte leakage (**D**) of sunflower cultivated under normal conditions and Cr toxicity i.e., 150 mg Cr/ kg soil (150Cr) and 200 mg Cr/ kg soil (200Cr). Bars are means of 4 replicates ± SE. Treatments were compared using Fisher’s LSD; p ≤ 0.05. Different letters on the bars showed significant changes. AMF = Arbuscular mycorrhizae fungi; ZQDB = zinc quantum dot biochar

The results revealed significant variations in the transpiration rates among the treatments. In the control group, the transpiration rate was measured at 7.03 mmol H_2_O m²/sec. Treatment with AMF + 0.40ZQDB resulted in a slightly increased transpiration rate of 7.84 mmol H_2_O m²/sec, representing a ~ 12% change. Intensifying the stress treatment to AMF + 0.80ZQDB further increased the transpiration rate to 8.10 mmol H_2_O m²/sec, corresponding to a ~ 15% change. The highest stress treatment, AMF + 1.20ZQDB, exhibited the most substantial impact on the transpiration rate, with a of 8.22 mmol H_2_O m²/sec and a percentage change of ~ 17%. Moreover, the study explored the effects of amendments application on the transpiration rate at different stress concentrations, namely 150Cr and 200Cr. The control group for the 150Cr concentration displayed a transpiration rate of 7.17 mmol H_2_O m²/sec. Treatment with AMF + 0.40ZQDB resulted in a slightly increased transpiration rate of 7.53 mmol H_2_O m²/sec, indicating a ~ 5% change. Treatment with AMF + 0.40ZQDB resulted in a transpiration rate of 6.77 mmol H_2_O m²/sec, corresponding to an ~ 8% change. Further stress treatment with AMF + 0.80ZQDB increased the transpiration rate to 7.11 mmol H_2_O m²/sec, indicating a 13.63% change. The highest stress treatment, AMF + 1.20ZQDB, for the 200Cr concentration yielded a of 7.28 mmol H_2_O m²/sec, representing a ~ 16% change (Fig. 4A).

For control, the stomatal conductance was measured at 712.68 mol/m²/s. Treatment with AMF + 0.40ZQDB resulted in a slightly increased stomatal conductance of 729.67 mol/m²/s, representing a ~ 2% change. Intensifying the stress treatment to AMF + 0.80ZQDB further increased the stomatal conductance to 737.37 mol/m²/s, corresponding to a ~ 4% change. The highest stress treatment, AMF + 1.20ZQDB, exhibited the greatest impact on stomatal conductance, with a of 744.49 mol/m²/s and a percentage change of ~ 5%. The effects of amendments application on stomatal conductance were also investigated at two additional stress concentrations, namely 150Cr and 200Cr. The control group for the 150Cr concentration displayed a stomatal conductance of 652.49 mol/m²/s. Treatment with AMF + 0.40ZQDB showed a slightly increased stomatal conductance of 675.28 mol/m²/s, indicating a ~ 4% change. Further stress treatment with AMF + 0.80ZQDB led to a stomatal conductance of 705.85 mol/m²/s, representing an ~ 8% change. The highest stress treatment, AMF + 1.20ZQDB, for the 150Cr concentration yielded a of 715.15 mol/m²/s, signifying a ~ 10% change. For the 200Cr concentration, the control group exhibited a stomatal conductance of 604.12 mol/m²/s. The highest stress treatment, AMF + 1.20ZQDB, for the 200Cr concentration yielded a of 641.70 mol/m²/s, representing a ~ 6% change (Fig. 4B).

Results showed that AMF + 0.40ZQDB resulted in a slightly increased chlorophyll content of 16.02 (SPAD), representing a ~ 6% change. Intensifying the stress treatment to AMF + 0.80ZQDB further increased the chlorophyll content to 16.46 (SPAD), corresponding to ~ 9% change. The highest stress treatment, AMF + 1.20ZQDB, exhibited the most significant impact on chlorophyll content, with a of 16.70 (SPAD) and a percentage change of ~ 10%. The effects of amendments application on chlorophyll content were also examined at two additional stress concentrations: 150Cr and 200Cr. The control group for the 150Cr concentration displayed a chlorophyll content of 14.43 (SPAD). Treatment with AMF + 0.40ZQDB resulted in a slightly increased chlorophyll content of 15.08 (SPAD), indicating a ~ 5% change. Further stress treatment with AMF + 0.80ZQDB led to a chlorophyll content of 15.45 (SPAD), representing a ~ 7% change. The highest stress treatment, AMF + 1.20ZQDB, for the 150Cr concentration yielded a of 15.69 (SPAD), signifying an ~ 9% change. For the 200Cr concentration, the control group exhibited a chlorophyll content of 12.58 (SPAD). Treatment with AMF + 0.40ZQDB resulted in a chlorophyll content of 13.63 (SPAD), corresponding to an ~ 8% change. Further stress treatment with AMF + 0.80ZQDB increased the chlorophyll content to 13.90 (SPAD), indicating a 10.54% change. The highest stress treatment, AMF + 1.20ZQDB, for the 200Cr concentration yielded a of 14.21 (SPAD), representing a ~ 13% change (Fig. 4C).

### Electrolyte leakage, oleic acid percentage, and linoleic acid

The control group (NoAMF + NoZQDB) exhibited a electrolyte leakage of 44.84%. When treated with AMF + 0.40ZQDB, the electrolyte leakage decreased to 42.60%, indicating a ~ -5% change. Intensifying the stress treatment to AMF + 0.80ZQDB further reduced the electrolyte leakage to 39.59%, corresponding to a ~ -12% change. The highest stress treatment, AMF + 1.20ZQDB, showed the most significant impact on membrane stability, with a electrolyte leakage of 35.62% and a ~ -21% change. Similarly, for the 150Cr concentration, the control group displayed a electrolyte leakage of 61.71%. The highest stress treatment, AMF + 1.20ZQDB, resulted in a electrolyte leakage of 48.95%, signifying a ~ -21% change. For the 200Cr concentration, the control group exhibited a electrolyte leakage of 74.06%. Treatment with AMF + 0.40ZQDB reduced the electrolyte leakage to 70.88%, corresponding to a ~ -4% change. Further stress treatment with AMF + 0.80ZQDB led to a electrolyte leakage of 68.47%, indicating a ~ -8% change. The highest stress treatment, AMF + 1.20ZQDB, yielded a electrolyte leakage of 65.77%, representing an ~ -11% change (Fig. 4D).

### Oleic acid, linoleic acid, palmitic acid and steric acid

**Fig. 5:**
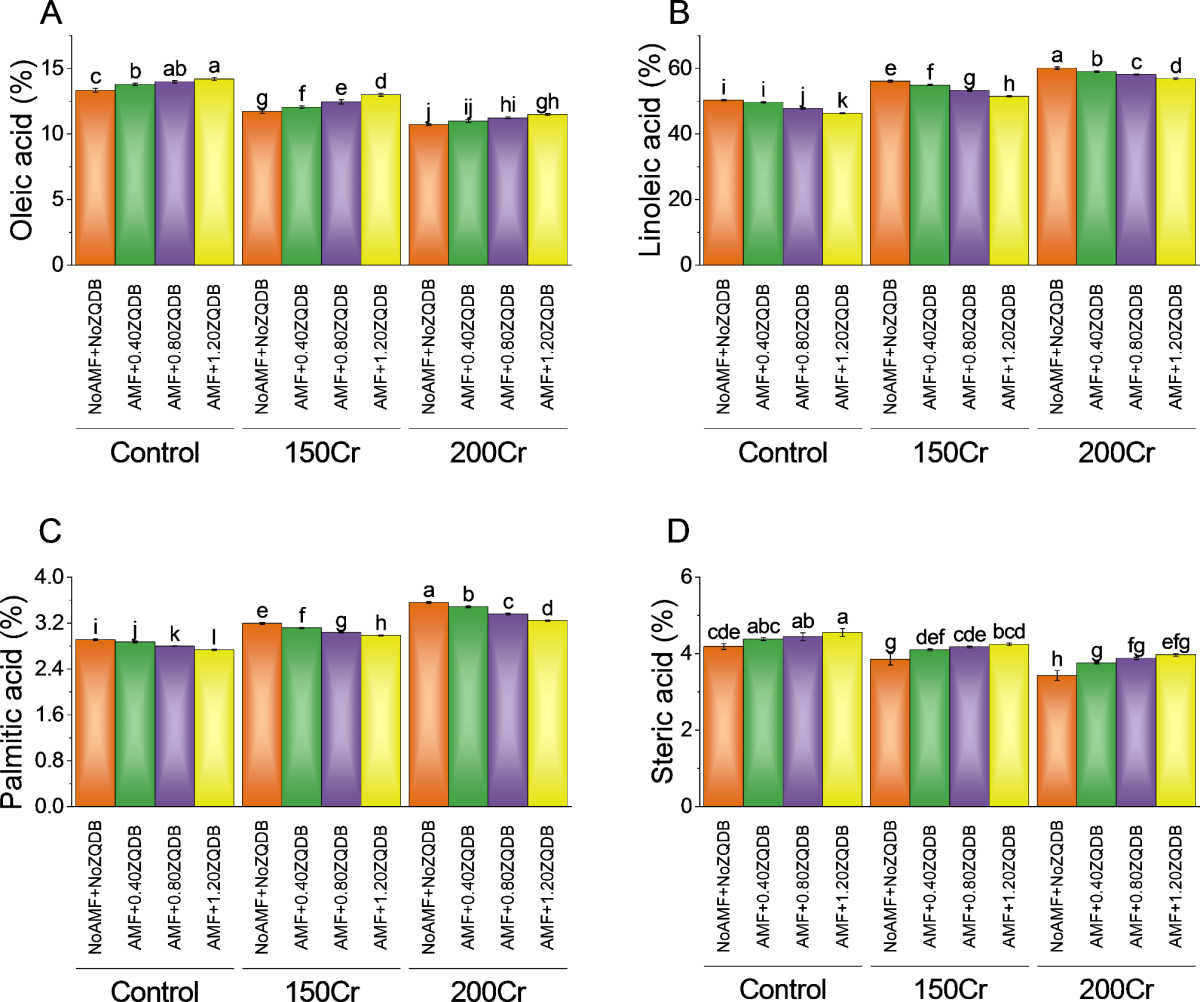
Effect of treatments on the oleic acid (**A**), linoleic acid (**B**), palmitic acid (**C**), and steric acid (**D**) of sunflower cultivated under normal conditions and Cr toxicity, i.e., 150 mg Cr/ kg soil (150Cr) and 200 mg Cr/ kg soil (200Cr). Bars are means of 4 replicates ± SE. Treatments were compared using Fisher’s LSD; p ≤ 0.05. Different letters on the bars showed significant changes. AMF = Arbuscular mycorrhizae fungi; ZQDB = zinc quantum dot biochar

The control group (NoAMF + NoZQDB) exhibited a oleic acid percentage of 13.33%. When treated with AMF + 0.40ZQDB, the oleic acid percentage increased to 13.79%, representing a ~ 3% change. Intensifying the stress treatment to AMF + 0.80ZQDB further elevated the oleic acid percentage to 13.99%, indicating a ~ 5% change. The highest stress treatment, AMF + 1.20ZQDB, resulted in a oleic acid percentage of 14.20%, corresponding to a ~ 7% change. Similarly, for the 150Cr concentration, the control group displayed a oleic acid percentage of 11.71%. The highest stress treatment, AMF + 1.20ZQDB, resulted in a oleic acid percentage of 12.99%, signifying a ~ 11% change. For the 200Cr concentration, the control group exhibited a oleic acid percentage of 10.73%. Treatment with AMF + 0.40ZQDB increased the oleic acid percentage to 11.00%, indicating a ~ 3% change. Further stress treatment with AMF + 0.80ZQDB led to a oleic acid percentage of 11.24%, representing a ~ 4% change. The highest stress treatment, AMF + 1.20ZQDB, yielded a oleic acid percentage of 11.48%, corresponding to a ~ 7% change (Fig. 5A).

When treated with AMF + 0.40ZQDB, the percentage decreased to 49.70%, representing a percentage change of ~ -1%. Intensifying the stress treatment to AMF + 0.80ZQDB further reduced the percentage of linoleic acid to 47.76%, indicating a percentage change of ~ -5%. The highest stress treatment, AMF + 1.20ZQDB, resulted in a linoleic acid percentage of 46.38%, corresponding to a percentage change of ~ -8%. Similarly, for the 150Cr concentration, the control group exhibited a linoleic acid percentage of 56.11%. Treatment with AMF + 0.40ZQDB reduced the percentage to 54.95%, indicating a percentage change of ~ -2%. Further stress treatment with AMF + 0.80ZQDB led to a linoleic acid percentage of 53.29%, representing a percentage change of ~ -5%. Treatment with AMF + 0.40ZQDB decreased the percentage to 59.05%, indicating a percentage change of ~ -2%. Further stress treatment with AMF + 0.80ZQDB led to a linoleic acid percentage of 58.16%, representing a percentage change of ~ -3%. The highest stress treatment, AMF + 1.20ZQDB, yielded a linoleic acid percentage of 56.83%, corresponding to a percentage change of ~ -5% (Fig. 5B).

Treatment with AMF + 0.40ZQDB resulted in a slightly decreased percentage of 2.87%, corresponding to a percentage change of ~ -1%. Further intensifying the stress treatment to AMF + 0.80ZQDB led to a palmitic acid percentage of 2.80%, indicating a percentage change of ~ -4%. The highest stress treatment, AMF + 1.20ZQDB, resulted in a palmitic acid percentage of 2.74%, representing a percentage change of ~ -6%. Similarly, for the 150Cr concentration, the control group exhibited a palmitic acid percentage of 3.20%. Treatment with AMF + 0.40ZQDB reduced the percentage to 3.12%, indicating a percentage change of ~ -2%. Further stress treatment with AMF + 0.80ZQDB led to a palmitic acid percentage of 3.04%, representing a percentage change of ~ -5%. The highest stress treatment, AMF + 1.20ZQDB, resulted in a palmitic acid percentage of 2.98%, signifying a percentage change of ~ -7%. For the 200Cr concentration, the control group displayed a palmitic acid percentage of 3.56%. Treatment with AMF + 0.40ZQDB decreased the percentage to 3.48%, indicating a percentage change of ~ -2%. Further stress treatment with AMF + 0.80ZQDB led to a palmitic acid percentage of 3.36%, representing a percentage change of ~ -6%. The highest stress treatment, AMF + 1.20ZQDB, yielded a palmitic acid percentage of 3.25%, corresponding to a percentage change of ~ -9% (Fig. 5C).

In the control group (NoAMF + NoZQDB), the percentage of stearic acid was 4.19%. Treatment with AMF + 0.40ZQDB resulted in a slightly increased percentage of 4.38%, corresponding to a percentage change of ~ 5%. Treatment with AMF + 0.40ZQDB increased the percentage to 4.10%, indicating a percentage change of ~ 6%. Further stress treatment with AMF + 0.80ZQDB led to a stearic acid percentage of 4.18%, representing a percentage change of ~ 8%. The highest stress treatment, AMF + 1.20ZQDB, resulted in a stearic acid percentage of 4.25%, signifying a percentage change of ~ 10%. For the 200Cr concentration, the control group displayed a stearic acid percentage of ~ 3%. Treatment with AMF + 0.40ZQDB increased the percentage to 3.76%, indicating a percentage change of ~ 10%. Further stress treatment with AMF + 0.80ZQDB led to a stearic acid percentage of 3.88%, representing a percentage change of ~ 13%. The highest stress treatment, AMF + 1.20ZQDB, yielded a stearic acid percentage of 3.97%, corresponding to a percentage change of ~ 16% (Fig. 5D).

### Antioxidants

**Fig. 6:**
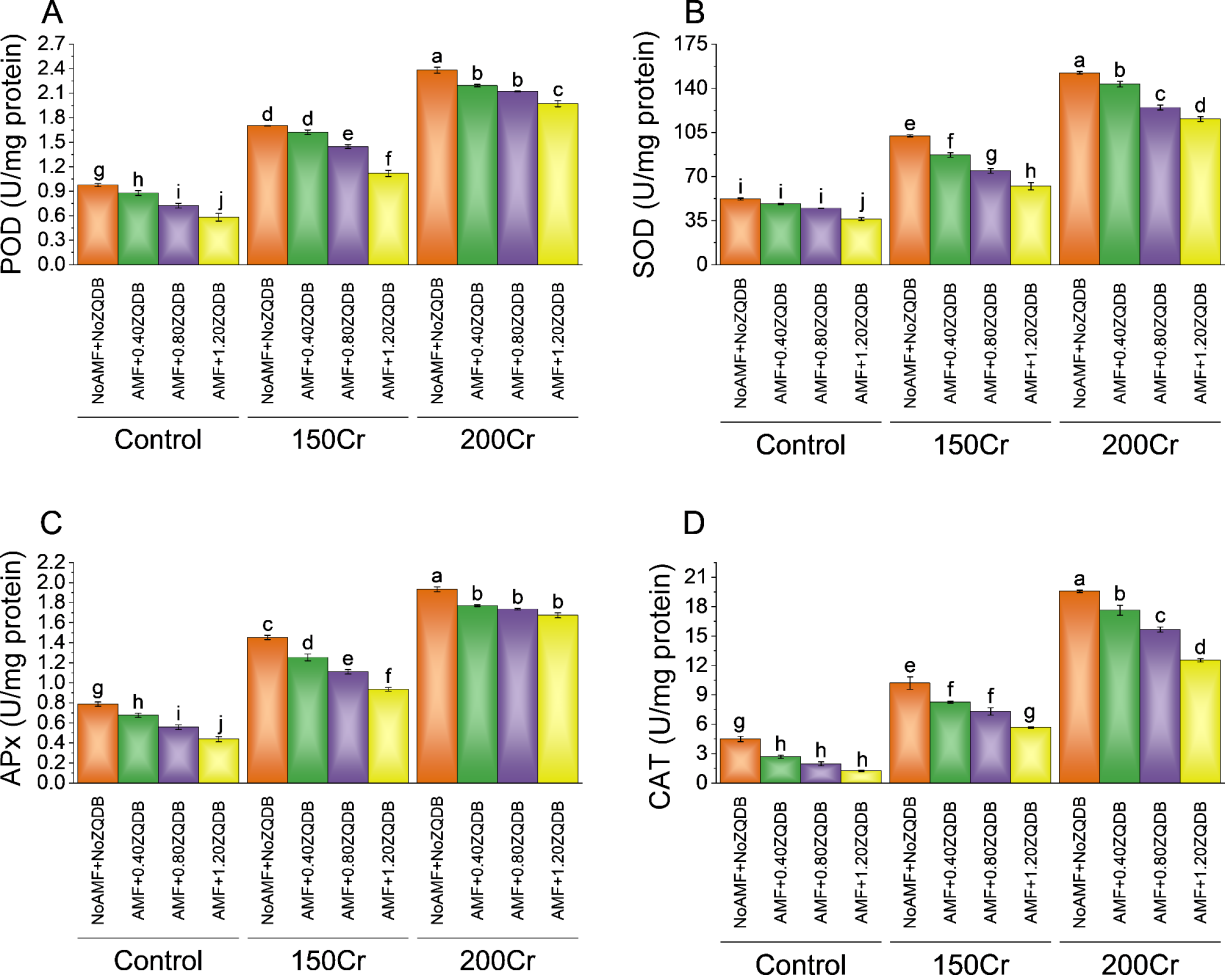
Effect of treatments on Peroxidase POD (**A**), Superoxidase (SOD) (**B**), APx (**C**), and Catalase (CAT) (**D**) of sunflower cultivated under normal conditions and Cr toxicity i.e., 150 mg Cr/ kg soil (150Cr) and 200 mg Cr/ kg soil (200Cr). Bars are means of 4 replicates ± SE. Treatments were compared using Fisher’s LSD; p ≤ 0.05. Different letters on the bars showed significant changes. AMF = Arbuscular mycorrhizae fungi; ZQDB = zinc quantum dot biochar

Without stress treatment (NoAMF + NoZQDB), the control group exhibited a POD activity of 2.38 U/mg protein. When 0.40ZQDB was combined with AMF, the POD activity decreased by 8.58% to 2.19 U/mg protein in the control group. Treatment 0.80ZQDB and 1.20ZQDB combined with AMF, the POD activity continued to decline, showing percentage decreases of ~ 12% and ~ 21%, respectively. In the case of 150Cr treatment without AMF and ZQDB (NoAMF + NoZQDB), the POD activity was 1.70 U/mg protein. However, when AMF and 0.40ZQDB were introduced, there was a ~ 5% decrease in POD activity to 1.62 U/mg protein compared to the treatment NoAMF + NoZQDB under 150Cr stress. More ~ 18% and ~ 52% reduction were recorded when 0.80ZQDB and 1.20ZQDB treatments were combined with AMF in 150Cr stress over control treatment (NoAMF + NoZQDB). In the highest stress treatment group, 200Cr without any AMF or ZQDB stress (NoAMF + NoZQDB) resulted in a POD activity of 0.98 U/mg protein. Adding 0.40ZQDB treatment with AMF led to an ~ 11% reduction in POD activity, bringing it down to 0.88 U/mg protein over the control (NoAMF + NoZQDB) under 200Cr stress. The most significant percentage decrease in POD activity was observed in the presence of AMF + 0.80ZQDB and AMF + 1.20ZQDB treatment, with reductions of ~ 35% and ~ 68%, compared to the control group (NoAMF + NoZQDB) in 200Cr stress (Fig. 6A).

In the control group without AMF and ZQDB treatment, the SOD activity was 152.26 U/mg protein. When AMF was applied with 0.40ZQDB, there was a ~ 6% decrease in SOD activity (143.26 U/mg protein). With 0.80ZQDB and AMF, SOD activity decreased by 22.11% (124.69 U/mg protein), and with 1.20ZQDB and AMF, there was a ~ 32% decrease (115.62 U/mg protein) in control in comparison to the NoAMF + NoZQDB treatment. In the 150Cr stress group without AMF and ZQDB treatment, SOD activity was 102.34 U/mg protein. Applying 0.40ZQDB and AMF resulted in a ~ 18% decrease in SOD activity (87.03 U/mg protein). In comparison, 0.80ZQDB and AMF led to a substantial ~ 38% decrease (74.42 U/mg protein) over the NoAMF + NoZQDB treatment in 150Cr stress. Treatment 1.20ZQDB, and AMF, caused a remarkable ~ 64% decrease in SOD activity (62.39 U/mg protein) relative to the control NoAMF + NoZQDB under 150Cr stress. For the 200Cr stress group without AMF and ZQDB treatment, SOD activity was 52.16 U/mg protein. Adding 0.40ZQDB and AMF led to an ~ 8% decrease in SOD activity (48.26 U/mg protein). In comparison, 0.80ZQDB and AMF resulted in a ~ 16% decrease (44.81 U/mg protein) from the NoAMF + NoZQDB in 200Cr stress. Treatment 1.20ZQDB and AMF in 200Cr stress showed a substantial ~ 44% decrease (36.30 U/mg protein) over the NoAMF + NoZQDB treatment (Fig. 6B).

In the control group without NoAMF + NoZQDB, the APx activity was 0.79 U/mg protein. When the control group was treated with AMF and 0.40ZQDB, the APx activity decreased to 0.68 U/mg protein, representing a ~ 16% decrease. With an increased concentration of 0.80ZQDB, the APx activity in the control group further decreased to 0.56 U/mg protein, marking a ~ 41% decrease. The highest concentration of 1.20ZQDB in the control group led to the most significant decrease, with APx activity at 0.44 U/mg protein, showing a ~ 80% decrease over the NoAMF + NoZQDB. In the 150Cr stress treatment group without AMF and ZQDB, the APx activity was 1.45 U/mg protein. When AMF and 0.40ZQDB were introduced, APx activity decreased to 1.25 U/mg protein, indicating a ~ 16% decrease in APx activity over the NoAMF + NoZQDB treatment in 150Cr stress. When AMF + 0.80ZQDB treatment was applied, APx activity in the 150Cr group decreased to 1.11 U/mg protein, representing a ~ 31% decrease from NoAMF + NoZQDB treatment. The highest concentration of AMF + 1.20ZQDB in the 150Cr group led to a further decrease, with APx activity at 0.93 U/mg protein, marking a ~ 55% decrease. In the 200Cr stress treatment group without AMF and ZQDB, the APx activity was 1.93 U/mg protein. When AMF and 0.40ZQDB were introduced, APx activity decreased to 1.77 U/mg protein, indicating a ~ 9% decrease. With treatment, AMF + 0.80ZQDB, APx activity in the 200Cr group decreased to 1.73 U/mg protein, representing an ~ 11% decrease in 200Cr stress. Treatment 1.20ZQDB with AMF in the 200Cr group led to a ~ 15% decrease, with a APx activity of 1.68 U/mg protein (Fig. 6C).

In the case of the 150Cr treatments, the catalase activity decreased activity compared to the control group (NoAMF + NoZQDB). The catalase activity was reduced to 8.26 U/mg protein (~ 23% decrease) when subjected to AMF and 0.40ZQDB, further declining to 7.31 U/mg protein (~ 40% decrease) with AMF and 0.80ZQDB, and significantly dropping to 5.68 U/mg protein (a substantial ~ 80% decrease) with AMF and 1.20ZQDB in comparison to the NoAMF + NoZQDB treatment in 150Cr stress. For the 200Cr treatments, the control group with NoAMF + NoZQDB treatment had a CAT activity 4.49 U/mg protein. However, when AMF and 0.40ZQDB treatment was applied, CAT activity decreased to 2.67 U/mg protein (~ 68% decrease) compared to the NoAMF + NoZQDB under 200Cr stress. With the addition of AMF + 0.80ZQDB, CAT activity was further reduced CAT activity to 1.95 U/mg protein, and with treatment AMF and 1.20ZQDB also showed a reduction in CAT activity under 200Cr stress (Fig. 6D).

#### GSH, H_2_O_2_ and MDA

**Fig. 7:**
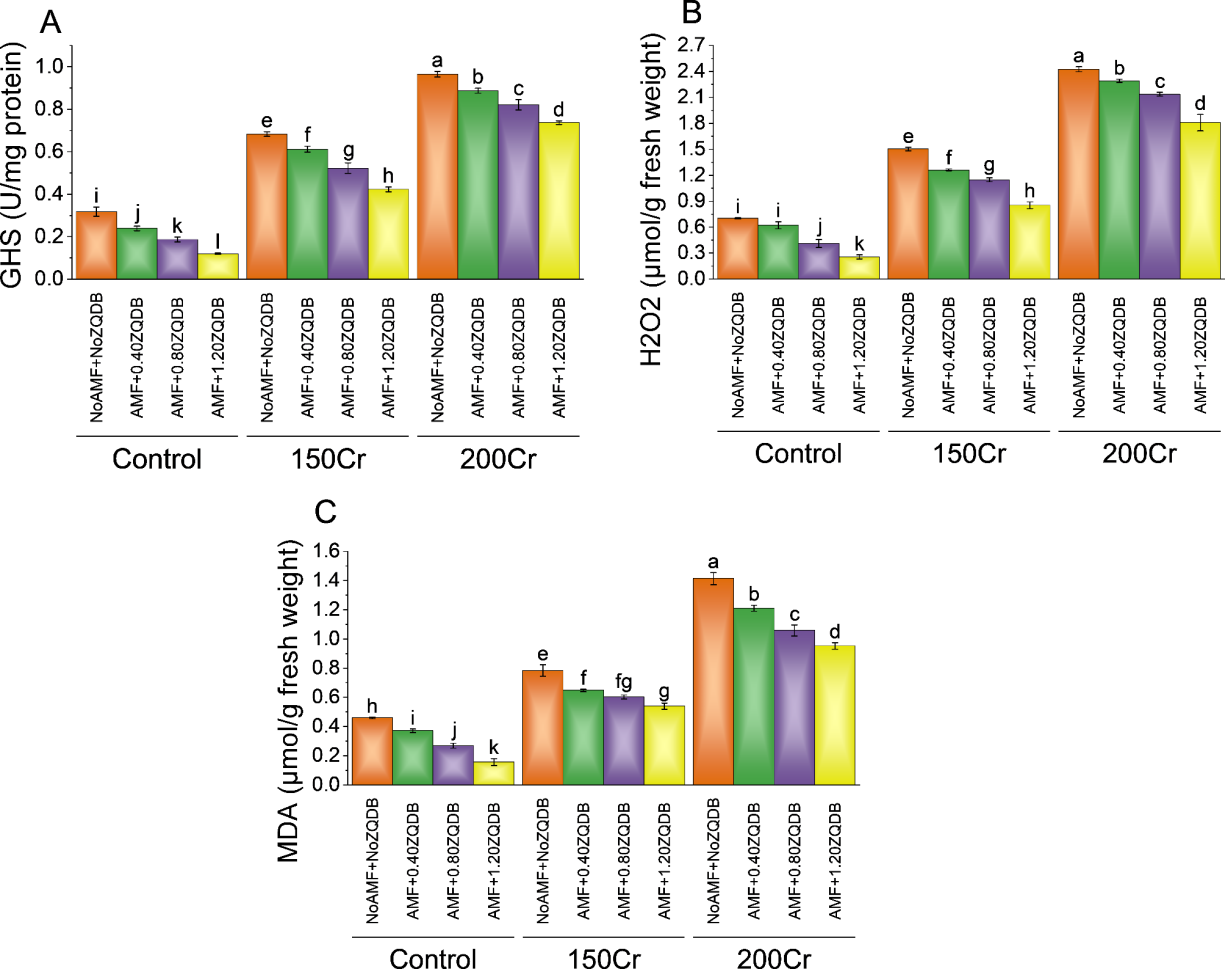
Effect of treatments on GSH (**A**), H_2_O_2_ (**B**), and MDA (**C**) of sunflower cultivated under normal conditions and Cr toxicity i.e., 150 mg Cr/ kg soil (150Cr) and 200 mg Cr/ kg soil (200Cr). Bars are means of 4 replicates ± SE. Treatments were compared using Fisher’s LSD; p ≤ 0.05. Different letters on the bars showed significant changes. AMF = Arbuscular mycorrhizae fungi; ZQDB = zinc quantum dot biochar

In the case of 150Cr, without AMF and ZQDB, it resulted in a glutathione (GSH) content of 0.68 U/mg protein, indicating a percentage change of ~ 115% compared to the control group. Introducing AMF with 0.40ZQDB and 0.80ZQDB further increased the GSH contents to 0.61 U/mg protein and 0.52 U/mg protein, corresponding to percentage changes of ~ 156% and ~ 180%, respectively. The highest GSH content of 0.42 U/mg protein was observed when AMF was combined with 1.20ZQDB, resulting in a remarkable percentage change of ~ 254%. The introduction of AMF with 0.40ZQDB and 0.80ZQDB resulted in GSH contents of 0.82 U/mg protein and 0.88 U/mg protein, corresponding to percentage changes of ~ 245 and ~ 374%, respectively. The combination of AMF and 1.20ZQDB yielded the highest GSH content of 0.97 U/mg protein and an exceptional percentage change of ~ 709% (Fig. 7A).

Introducing AMF with 0.40ZQDB and 0.80ZQDB decreased the H_2_O_2_ contents to 1.26 µmol/g fresh weight and 1.15 µmol/g fresh weight, corresponding to percentage changes of ~ 103% and ~ 180%, respectively. The lowest H_2_O_2_ content of 0.85 µmol/g fresh weight was observed when AMF was combined with 1.20ZQDB, resulting in a significant percentage change of ~ 233%. The introduction of AMF with 0.40ZQDB and 0.80ZQDB resulted in H_2_O_2_ contents of 2.14 µmol/g fresh weight and 2.29 µmol/g fresh weight, corresponding to percentage changes of ~ 244% and ~ 459%, respectively. The combination of AMF and 1.20ZQDB yielded the highest H_2_O_2_ content of 2.42 µmol/g fresh weight and an exceptional percentage change of ~ 847% (Fig. 7B).

Under the control stress treatment, the MDA content was measured at 0.46 µmol/g fresh weight. When subjected to the NoAMF + 0.40ZQDB treatment, the MDA content decreased by ~ 24% to 0.37 µmol/g fresh weight. For the AMF + 0.80ZQDB treatment, there was a more significant reduction in MDA content, with a ~ 71% decrease over the control, resulting in a of 0.27 µmol/g fresh weight. The most substantial decrease in MDA content occurred with the AMF + 1.20ZQDB treatment, which showed a remarkable reduction from the control, with a value of 0.16 µmol/g fresh weight. In the case of the 150Cr stress treatment, the MDA content in the control group was 0.78 µmol/g fresh weight. Under the AMF + 0.40ZQDB treatment, MDA content decreased by ~ 21% to 0.65 µmol/g fresh weight. The AMF + 0.80ZQDB treatment resulted in a ~ 30% decrease, with an MDA content of 0.60 µmol/g fresh weight than the control treatment. The AMF + 1.20ZQDB treatment showed a substantial ~ 45% reduction in MDA content, measuring 0.54 µmol/g fresh weight. For the 200Cr stress treatment, the control group had an MDA content of 1.41 µmol/g fresh weight. In the AMF + 0.40ZQDB treatment, MDA content decreased by ~ 17% to 1.21 µmol/g fresh weight in contrast to the NoAMF + NoZQDB under 200 Cr stress. A more significant reduction of ~ 34% was observed under the AMF + 0.80ZQDB treatment related to the NoAMF + NoZQDB, resulting in an MDA content of 1.06 µmol/g fresh weight in 200Cr stress. The AMF + 1.20ZQDB treatment displayed the most substantial reduction in MDA content in 200cr stress, with a ~ 49% decrease from the control (NoAMF + NoZQDB), resulting in a MDA content of 0.95 µmol/g fresh weight (Fig. 7C).

### Oil and protein contents

**Fig. 8:**
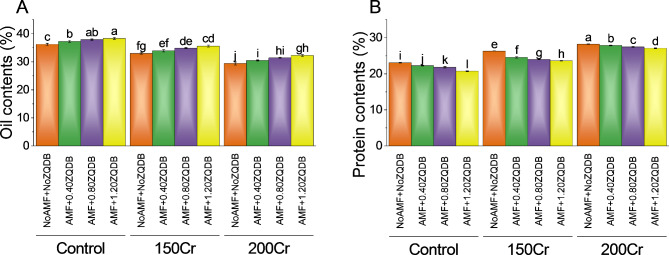
Effect of treatments on oil contents (**A**) and protein contents (**B**) of sunflower cultivated under normal conditions and Cr toxicity i.e., 150 mg Cr/ kg soil (150Cr) and 200 mg Cr/ kg soil (200Cr). Bars are means of 4 replicates ± SE. Treatments were compared using Fisher’s LSD; p ≤ 0.05. Different letters on the bars showed significant changes. AMF = Arbuscular mycorrhizae fungi; ZQDB = zinc quantum dot biochar

In the 150Cr treatment group, the absence of AMF and ZQDB resulted in oil content of 33.02%, representing a percentage change of ~ -9% compared to the control group. When AMF was combined with 0.40ZQDB and 0.80ZQDB, the oil contents increased slightly to 33.94% and 34.88%, respectively, with percentage changes of ~ -9% and ~ -8%. The highest oil content of 35.61% was observed when AMF was combined with 1.20ZQDB, indicating a percentage change of ~ -7%. Upon increasing the Cr concentration to 200Cr, the control group exhibited a significantly lower oil content of 29.41%, resulting in a percentage change of ~ -19% compared to the initial control value. The combination of AMF and 1.20ZQDB yielded the highest oil content of 32.25%, with a percentage change of ~ -16% (Fig. 8A).

For 150Cr, the absence of AMF and ZQDB led to a protein content of 26.32%, representing a percentage change of ~ 14% compared to the control group. Combining AMF with 0.40ZQDB and 0.80ZQDB resulted in slightly lower protein contents of 24.54% and 23.98%, corresponding to percentage changes of ~ 10% and ~ 10%, respectively. The introduction of AMF with 0.40ZQDB and 0.80ZQDB resulted in protein contents of 27.85% and 27.46%, corresponding to percentage changes of ~ 25% and ~ 26%, respectively. The combination of AMF and 1.20ZQDB yielded a protein content of 27.08% and a substantial percentage change of ~ 31% (Fig. 8B).

**Fig. 9:**
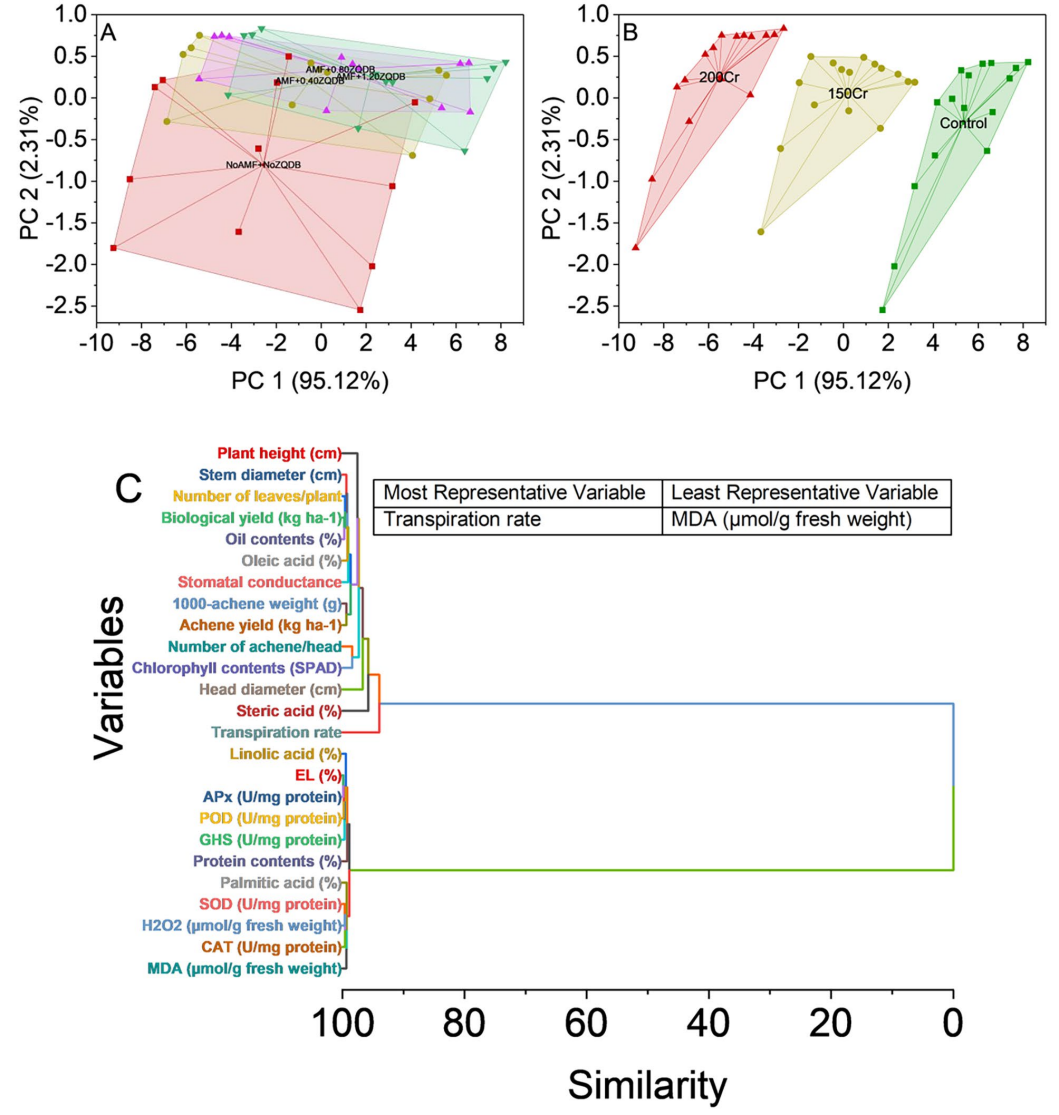
Cluster plot with convex hull for treatments (**A**), Cr stress (**B**), and hierarchical cluster plot for studied attributes (**C**)

The analysis of the convex hull representation of data points in a multidimensional space, defined by PC1 and PC2, under various treatment conditions provides valuable insights into the distribution and variation of the data. The results indicate distinct patterns associated with different treatments, shedding light on the effects of these treatments on the data points. Under the NoAMF + NoZQDB treatment, the convex hull encompasses a cluster of data points, suggesting a relatively tight data grouping. This may indicate a certain level of homogeneity or similarity among samples subjected to this treatment. In contrast, under the AMF + 0.40ZQDB treatment, the convex hull expands, encompassing a broader range of data points. This expansion implies greater variability in data and potentially more pronounced differences between samples within this treatment group. Similar trends can be observed for the AMF + 0.80ZQDB and AMF + 1.20ZQDB treatments (Fig. 9A).

The analysis of the convex hull representation of data points in a multidimensional space defined by PC1 and PC2 under different stress conditions reveals distinct patterns associated with varying stress levels. This method offers valuable insights into the distribution and arrangement of data points, helping to discern how stress conditions impact the data. Under the Control stress condition, the convex hull comprises a cluster of data points, suggesting a relatively tight grouping and a certain degree of similarity among samples subjected to this condition. The proximity of data points within the convex hull indicates that they share common characteristics, possibly reflecting a baseline or normal state in the study context. In contrast, the convex hulls for the 150Cr and 200Cr stress conditions reveal a more extensive distribution of data points. This broader expansion signifies increased variability in the data. It suggests that applying chromium stress results in more pronounced differences among samples. The expansion of the convex hulls for these stress conditions implies that stress may disrupt or alter the underlying patterns in the data, potentially reflecting the physiological responses of the system to the stressors (Fig. 9B).

The analysis indicates several variables exhibit close similarity, forming clusters at different hierarchy levels. For example, electrolyte leakage (%) and APx (U/mg protein) are highly similar, with a similarity score of 0.15291, indicating a potential connection between electrolyte leakage and ascorbate peroxidase activity. Another cluster includes POD (U/mg protein), suggesting that peroxidase activity is related to these variables. A separate cluster involves biological yield (kg ha^− 1^) and oil contents (%), highlighting a possible connection between these agricultural yield-related parameters. Additional clusters involve variables related to enzymatic activities such as GHS (U/mg protein), SOD (U/mg protein), and H_2_O_2_ (µmol/g fresh weight). This suggests that these parameters may share underlying factors influencing their behaviour. The analysis also reveals clusters related to plant growth and development. For instance, number of leaves/plant and stem diameter (cm) form a cluster, indicating a potential relationship between these growth-related characteristics. Variables related to fatty acid composition, such as linolic acid (%), palmitic acid (%), and oleic acid (%), appear in clusters together, suggesting common factors affecting the composition of these fatty acids in the plant. Moreover, variables linked to physiological and yield-related attributes, including number of achene/head, chlorophyll contents (SPAD), plant height (cm), and head diameter (cm), form clusters, indicating associations between these attributes. The hierarchical cluster analysis culminates in a final cluster consisting of variables with high similarity scores. These variables, transpiration rate, steric acid (%), transpiration rate, and stomatal conductance, are grouped closely, indicating they may share common influences and responses (Fig. 9C).

## Discussion

The present study investigated the sensitivity of sunflower plants to Cr toxicity. It explored the potential mitigating effects of AMF and ZQDB applications. We assessed a range of growth and yield attributes and physiological responses to understand the impact of Cr stress and the potential benefits of AMF and ZQDB treatments. Our results revealed a significant sensitivity of sunflower plants to increasing Cr concentrations. As the Cr concentration increased, various growth and yield attributes of the sunflower plants showed a declining trend. The most pronounced effect was observed at the highest Cr application rate of 200 mg kg^− 1^, where adverse impacts on growth and yield parameters were most prominent. The application of AMF and ZQDB demonstrated a synergistic effect in enhancing the sunflower plant’s ability to survive and increase under conditions of Cr toxicity. AMF is crucial in facilitating nutrient uptake, including essential elements required for plant growth. By forming a symbiotic relationship with sunflower roots, AMF improved nutrient availability and absorption, thereby mitigating the negative effects of chromium toxicity on plant height [17]. This finding aligns with prior research on the beneficial role of AMF in nutrient acquisition and plant growth [15]. Zinc quantum dot biochar, known for its porous structure, improved plant height by promoting nutrient availability in the soil. Biochar capacity to retain essential nutrients and release them gradually over time ensures a continuous supply of nutrients for sunflower plants [7]. The sustained nutrient availability facilitated optimal growth and development, ultimately increasing plant height. This observation is consistent with studies highlighting the positive impact of biochar on soil properties and plant growth [20]. AMF enhanced nutrient uptake and improved root development and branching, resulting in an increased root surface area and nutrient absorption capacity [34, 35]. These effects translated into stronger and thicker stems in sunflowers under chromium toxicity. An improved root system facilitated efficient nutrient and water transport, increasing diameter [36]. The ability of ZQDB to improve soil structure and water-holding capacity further supported the growth of sunflower plants by promoting robust stem development and, consequently, an increased stem diameter [37]. The increased nutrient uptake, facilitated by the combination of AMF and ZQDB, positively impacted the reproductive growth of sunflowers, particularly head diameter [9]. Nutrients like phosphorus, essential for flower development and size, were made more available and efficiently absorbed through the synergistic action of AMF and biochar [38]. This enhanced nutrient supply contributed to larger, well-developed sunflower heads [39]. Chromium toxicity disrupts cellular membranes, increasing electrolyte leakage from plant cells [38, 40]. AMF and ZQDB could reduce electrolyte leakage by improving cellular membrane integrity in sunflowers under chromium stress [1]. According to the hierarchical cluster analysis (Fig. 9C), transpiration rate is the most representative parameter contributing more to enhancing plant growth, and MDA is the least represented parameter. It’s because transpiration rate is crucial in plant gas exchange, particularly in sunflowers, where water is absorbed, transported, and released through stomata in leaves. Transpiration regulates plant temperature by releasing water vapour, which is beneficial under stress conditions like chromium toxicity and essential for maintaining physiological processes [31]. Transpiration is intimately linked to gas exchange, including the uptake of carbon dioxide (CO_2_) and the release of oxygen (O_2_) through stomatal openings [41]. This gas exchange is vital for photosynthesis, the process through which plants produce energy and organic compounds. Enhanced transpiration can improve photosynthesis, supporting growth and yield [44]. Second, MDA, a compound formed when cellular lipids are damaged by ROS, is a significant indicator of oxidative stress, which is generally detrimental to plant health, as it is often caused by stress conditions [35]. High MDA level can cause impaired cell membranes and damage, hindering plant growth. AMF enhances plant antioxidant systems, protects against oxidative stress, and contributes to maintaining cellular membrane integrity [42, 43]. ZQDB ability to reduce heavy metal bioavailability further mitigated the direct toxic effects of chromium on cellular membranes. In response to chromium stress, sunflowers exhibited increased POD and APx activity, indicative of their defense mechanisms against oxidative damage [36]. AMF plays a significant role in enhancing the antioxidant defense mechanisms of sunflowers by regulating enzymes like POD, CAT, APx, and SOD [44]. These antioxidants contributed to the detoxification of ROS and the mitigation of oxidative damage. ZQDB, by reducing chromium uptake and its negative impact on membrane stability, potentially minimized electrolyte leakage in sunflower plants [45]. The present study also observed reduced oil contents in sunflower seeds due to chromium stress, which can be attributed to disruptions in metabolic processes and lipid metabolism caused by chromium toxicity [41]. On the contrary, an increase in protein content was noted in sunflowers under chromium stress, indicating the activation of stress-responsive genes involved in protein synthesis and accumulation [42, 43]. These findings align with previous research highlighting the response of plants to chromium stress, where oxidative stress induces changes in both oil and protein contents [46].

## Conclusions

AMF, along with 1.20%ZQDB, was an effective amendment for mitigating Cr stress in sunflower plants. The treatment showed great potential to improve the growth, gas exchange attributes, chlorophyll contents, and yield attributes of sunflowers under chromium toxicity. Growers are recommended to add AMF + 1.20%ZQDB to achieve better sunflower growth in Cr-toxic soils. More investigations are suggested on different crops to declare AMF + 1.20%ZQDB as the best amendment against Cr toxicity in different soil textures.

## Data Availability

All data generated or analyzed during this study are included in this published article.

## Declarations

### Study protocol must comply with relevant institutional, national, and international guidelines and legislation

Our experiment follows the with relevant institutional, national, and international guidelines and legislation.
